# Are We Following MHRA Guidance Regarding Valproate Prescription in Women of Child-Bearing Potential?

**DOI:** 10.1192/bjo.2022.487

**Published:** 2022-06-20

**Authors:** Adarsh Shetty, Joseph Polson

**Affiliations:** 1 Cwm Taf Morgannwg University Health Board, Merthyr Tydfil, United Kingdom; 2 Hywel Dda University Health Board, Carmarthen, United Kingdom

## Abstract

**Aims:**

To clarify if MHRA guidance regarding valproate prescription in women of childbearing potential is being followed by psychiatrists in Cwm Taf Morgannwg University Health Board.

**Methods:**

Women of childbearing potential who were prescribed valproate for mental health conditions were identified by contacting GP practice pharmacists. The notes of these patients were reviewed to see if the ARAF (Annual Risk Acknowledgement Form) had been completed. The GP records were cross-checked to see if highly effective contraception was prescribed for women who were on valproate. The first audit was done in November 2018, while the second audit was completed in December 2020. The main intervention after the first audit was general awareness raising amongst psychiatrists in secondary care about the MHRA guidance and the need for annual reviews, through email reminders and posters. The 2020 audit gathered detailed clinical information, including the reasons for prescribing valproate and the doses prescribed.

**Results:**

2018 – out of 53 women on valproate, 1 had a completed ARAF, and 15 were on highly effective contraception.

2020 – out of 48 women on valproate, none had a completed ARAF, and 13 were on highly effective contraception.

Concerningly, only half (46%) of these women were prescribed valproate for bipolar disorder. The rest were prescribed valproate for a variety of diagnoses including schizophrenia, cyclothymia, emotionally unstable personality disorder, and complex PTSD.

**Conclusion:**

Raising general awareness about MHRA guidance failed as an intervention in this audit. Hence, after the second audit, specific targeted emails are being sent to each sector's consultant psychiatrists, with a list of female patients of childbearing potential in their sector who are prescribed valproate. A valproate register was created for the Merthyr/Cynon and Rhondda/Taff Ely localities – to our knowledge, this is the first time this has been developed in Wales. The impact of these interventions is being evaluated with a third audit which is being done in March 2022. This audit cycle highlighted significant challenges in sharing information across primary and secondary care. Detailed information about patients on valproate, with information on prescribed contraception, was available only for the 2020 audit, due to the appointment of a pharmacist working across primary and secondary care.

